# Mannose binding lectin plays a crucial role in innate immunity against yeast by enhanced complement activation and enhanced uptake of polymorphonuclear cells

**DOI:** 10.1186/1471-2180-8-229

**Published:** 2008-12-18

**Authors:** Eveline C van Asbeck, Andy IM Hoepelman, Jelle Scharringa, Bjorn L Herpers, Jan Verhoef

**Affiliations:** 1 Eijkman-Winkler Institute for Medical & Clinical Microbiology, Utrecht University Hospital, Utrecht, the Netherlands; 2 Department of Internal Medicine & Infectious Diseases, Utrecht University Hospital, Utrecht, the Netherlands; 3 Department of Medical Microbiology & Immunology, St. Antonius Hospital Nieuwegein, the Netherlands

## Abstract

**Background:**

Mannose binding lectin (MBL) is an important host defence protein against opportunistic fungal pathogens. This carbohydrate-binding protein, an opsonin and lectin pathway activator, binds through multiple lectin domains to the repeating sugar arrays displayed on the surface of a wide range of clinically relevant microbial species. We investigated the contribution of MBL to antifungal innate immunity towards *C. parapsilosis in vitro*.

**Results:**

High avidity binding was observed between MBL and *C. albicans *and *C. parapsilosis*. Addition of MBL to MBL deficient serum increased the deposition of C4 and C3b and enhanced the uptake of *C. albicans*, *C. parapsilosis *and acapsular *C. neoformans *by polymorphonuclear cells (PMNs). Compared to other microorganisms, such as *Escherichia coli*, *Staphylococcus aureus *and *Cryptococcus neoformans*, *C. parapsilosis *and *Candida albicans *were potent activators of the lectin pathway.

**Conclusion:**

Our results suggest that MBL plays a crucial role in the innate immunity against infections caused by yeast by increasing uptake by PMN.

## Background

Mannose binding lectin [1], a plasma protein of hepatic origin that belongs to the family of calcium-dependent collagenous lectins (collectin), is an important protein of the innate immune system [1-6]. This carbohydrate-binding protein binds mannose and N-acetylglucosamine (GlcNAc) sugars and their derivates present on the surface of a wide range of clinically relevant microbial species and has the ability to distinguish self from nonself [4,5,7,8].

MBL initiates the lectin pathway of complement using attached mannose binding lectin-associated serine proteases (MASP-2) in an antibody- and C1q-independent manner [5,7,9]. MASP-2 is indistinguishable in specificity from the convertases found in the classical and alternative of complement activation and permits cleavage of C4 and C2 to form a C3 convertase [3,5,7,10]. Once it has bound, MBL is able to deploy a variety of anti-microbial activities, such as microbial opsonization and/or microbial lysis via membrane attack complexes [8,11]. However, it is unclear whether MBL acts as a direct opsonin or is merely enhances other complement pathways and/or antibody-mediated phagocytosis [5].

MBL deficiency, due to variation in the MBL gene, is one of the most common immunodeficiencies [5,12] and is associated with impaired phagocytosis by polymorphonuclear leukocytes and with an increased burden of infections, especially in immunocompromised individuals [13-15].

The clinically relevant opportunistic microorganism *C. parapsilosis *is now the second or third most common cause of systemic fungal infections after *C. albicans *[16-19]. It is especially prevalent in very low birth weight neonates, transplant patients, post-surgical patients, patients receiving intravenous hyperalimentation and patients with indwelling invasive devices [20-23]. Most patients at risk have some degree of immunosuppression. MBL has been shown to play a role in the first-line defence against *C. albicans *[9]. The fungal cell wall, which consists mainly of polymers of *N*-acetylglucosamine (chitin), glucose (β-glucan) and mannose (mannan) [15,24] is a candidate ligand for MBL and may be capable of activating the lectin complement pathway.

In this study we evaluated the role of MBL in the opsonophagocytosis of *C. parapsilosis*. MBL was found to be a crucial opsonin for optimal phagocytosis of *C. parapsilosis*, *C. albicans *and acapsular *C. neoformans*. Sera of patients with MBL deficiency have decreased opsonic capacity.

## Methods

### Microbial strains

A clinical isolate of *Candida parapsilosis *strain 05–173 (California Institute for Medical Research, San Jose, CA), as a reference *Candida albicans *strain ATCC 14053 (American Type Culture Collection), the thinly (<0.5 mm) encapsulated *Cryptococcus neoformans *strain NIH 37 (National Institute of Health, Bethesda, MD) and an acapsular mutant of *C. neoformans*, CAP 67 (E.S. Jacobson, Medical College of Virginia) *Staphylococcus aureus *Mu 50 (Japanese Collection of Staphylococcus Cultures (JCSC)), *S. aureus *KV 39 and KV 68 (clinical isolates from University Medical Centre Utrecht), *Escherichia coli *ATCC 25922 and *E. coli *ATCC 35218 (American Type Culture Collection) were used. In addition, *Saccharomyces cerevisiae *was used as a standard reference for the functional MBL test. Isolates were stored at -80°C in 40% glycerol. Before tests were performed, yeast strains were cultured overnight at 35°C on Sabouraud Dextrose Agar (SDA) and bacterial strains were cultured overnight at 37°C on blood agar and then kept at 4°C.

### Binding of MBL

Microorganisms (2 × 10^7 ^cells/mL) were incubated with 5 μg/mL of purified human MBL (90%) (HSR 003; Staten Serum Institut; MBL was purified in a two-step process by, affinity chromatography and gel filtration, with selecting for functionally active and oligomeric MBL. MASPs remain associated and co-elute with MBL [6]) in a total volume of 50 μL of veronal-buffered saline pH 7.4, containing Ca^2+ ^and Mg^2+ ^plus 0.05% BSA (VSB^2+^), on a shaking plate (150 rev/min) at 37°C for 30 min. Organisms were spun down for 5 min at 15000 rpm and the pellets were washed with VSB^2+ ^before suspension with mouse anti-MBL monoclonal antibody (mAbs) (10 μg/mL in VSB^2+^; HYB131-010; Antibody shop, Staten Serum Institute, Copenhagen, Denmark). After a 30-min incubation on ice, samples were centrifuged and washed as described above and were resuspended in FITC-labelled goat anti mouse IgG (DakoCytomation, Glostrup, Denmark) (80 μg/mL in PBS) and incubated on ice for 30 min. Suspensions were centrifuged and washed as described above. Samples were analyzed by flow cytometry (FACSCaliber, Becton Dickinson; Mountain View, CA) with measurement of mean fluorescence intensity (MFI). Experiments were done in duplicate and repeated at least three times. Negative controls were established for MBL binding by the omission of MBL. In order to evaluate whether the binding observed by C-type lectin interactions, inhibition experiments using a calcium chelating agent 20 mmol/L EDTA was added to the MBL solution 5 min before the addition of MBL to the microorganisms.

### Deposition of C4, and C3b

MBL-deficient serum was obtained from a subject who was homozygous for the LYPB haplotype of the MBL gene and had undetectable levels of serum MBL (< 0.05 μg/mL). Serum IgG was depleted from the MBL-deficient serum using a HiTrap Protein G column (GE Healthcare, Uppsala, Sweden) [25]. The freshly prepared MBL-deficient serum samples were aliqouted and stored at -70°C until use. Informed consent was obtained from the donor.

Microorganisms (2 × 10^6 ^cells/mL) were incubated in 50 μl HBS^2+ ^(Hepes-buffered saline, 20 mM Hepes, 140 mM NaCl, 5 mM CaCl_2 _and 2.5 mM MgCl_2_) containing 10% MBL-deficient serum supplemented with anti-C1q mAb (50 μg/mL; Sanquin, Amsterdam, The Netherlands) to inhibit the classical pathway [26] and with or without purified human MBL (2.5 μg/mL), in a sterile 96 well plate incubated for 2, 5, 15 and 30 minutes at a shaking plate (150 rev/min) at 37°C. The reaction in each well was stopped by adding 150 μL of ice-cold PBS. Suspensions were washed and centrifuged for 10 min at 3500 rpm. The supernatants were removed, and the pellets were suspended with 50 μL of a solution of murine monoclonal anti-human C4d (Quidel, San Diego, CA) (4 μg/mL in PBS). After a 30-min incubation on ice, the samples were centrifuged and washed as described above and the pellets were resuspended in FITC-labelled goat anti-mouse IgG (DakoCytomation, Glostrup, Denmark) (80 μg/mL in PBS) and were incubated on ice for 30 min. Suspensions were centrifuged and washed as described above and measured by flow cytometry. C4d deposition was evaluated in duplicate and repeated at least three times.

C3b deposition was analyzed by incubation of the organisms with 10% MBL-deficient sera in HBS^2+^, after which surface-bound C3b was detected with FITC-conjugated (Fab')_2 _anti-human C3 (Protos Immunoresearch, San Francisco, CA) (20 μg/mL in PBS). Detection of C3b deposition was the same as for deposition of C4d.

### Preparation of Polymorphonuclear Leukocytes

Human polymorphonuclear (PMN) cells were isolated from the blood of healthy volunteers using a Ficoll/Histopaque gradient with sodium heparin as anticoagulant (Greiner, Alphen a/d Rijn, The Netherlands) as described previously [27]. In brief, heparinized blood was diluted with an equal volume of PBS (pH 7.4), layered onto a gradient of Ficoll-Paque PLUS (GE Healthcare, Uppsala, Sweden) and Histopaque-1119 (Sigma-Aldrich, Steinheim, Germany), and centrifuged for 20 minutes at 400 × *g*. Neutrophils were collected from the Histopaque layer and washed with RPMI 1640 containing 25 mM Hepes (N-2-hydroxyethylpiperazine-N'-2-ethanesulfonic acid), L-glutamine (BioWhittaker, Walkerswille, MD) and 0.05% human serum albumin (Sanquin, Amsterdam, The Netherlands) (RPMI/HSA). The neutrophils were then subjected to a hypotonic shock with water for 30 s to lyse remaining erythrocytes.

### Fluorometric phagocytosis assay

Phagocytosis was performed using Fluorescein isothiocyanate (FITC)-labelled microorganisms, MBL-deficient serum and freshly isolated human neutrophils. In brief, organisms were mixed (100/100: vol/vol) with FITC (Sigma-Aldrich, Steinheim, Germany) (1 mg/mL in 1 M sodium carbonate buffer, pH 9.6) and incubated at 37°C for 1 h under constant shaking at 200 rpm. Organisms were washed with RPMI 1640 medium twice. For each separate experiment, organisms were cultured and labelled with FITC.

Aliquots of FITC-labelled microorganisms (50 μl of 2 × 10^6^cells/mL) were transferred in 96-wells microtiter plates. The pellets were tumbled with 20% MBL-deficient serum in the presence of 100 μg/mL anti-C1q mAb, with or without purified human MBL (5 μg/mL, final concentration) and incubated for 15 min on a shaking plate at 37°C (150 rev/min). The pellet of the pre-opsonised organisms was suspended in 50 μL of RPMI 1640 and incubated with 50 μL purified PMNs. Phagocytosis was stopped after 15 min by addition of 250 μL ice cold RPMI 1640 and the plate was centrifuged at 4°C at 1200 rpm for 10 min. Allophycocyanin (APC)-conjugated CD11b MAb (Becton Dickinson, San Jose, CA) served as a marker for human PMN [28] for the phagocytosis of yeast. The pellet from each well was suspended in 5 μL (250 μg/ml in PBS) and was incubated at 4°C for 30 min, followed by the addition of 250 μL of RPMI 1640 to each well and centrifugation at 4°C at 1200 rpm for 10 min. The cell pellet was suspended in 250 μL RPMI 1640 supplemented with 1% paraformaldehyde solution (PFA) and stored on ice for 30 min. Analysis of the samples was performed with a flow cytometer. Phagocytosis of the FITC-labelled microorganisms was evaluated by determining the proportion of labelled PMNs expressed as a percentage of the total population of PMNs. The maximum percentages, obtained within an experiment were assigned a value of 100% with all other percentages within the same experiment expressed as a relative percentage of this maximum. Negative controls for opsonophagocytosis were established by incubating organisms in RPMI 1640 containing neither serum nor purified human MBL in the opsonization step.

### Haemolytic MBL assay

A haemolytic MBL assay was used to study MBL activation by different microorganisms. This assay was previously described by Kuipers et al [3] and makes use of micro-organism-induced MBL activation in a dilution series of pooled human serum, followed by subsequent C5b-6-mediated bystander haemolysis of chicken erythrocytes. As a surplus of all down stream components of the lectin pathway are provided by a standardized concentration of MBL-deficient serum in this assay, the complement activation by bound MBL is the rate limiting step.

In brief, different microbial concentrations were added in 50 μl per well in a 96 well microtiter plate, and serially diluted in vertical rows (two-fold dilutions). As the source of MBL, human pooled serum (HPS, stored in aliquots as previously described) from healthy workers of our laboratory was diluted 1:32 in VSB^2+ ^and 50 μg/mL anti-C1q mAb [26], incubated on ice for 15 minutes, and then serially diluted (10^-0.5^) in VSB^2+ ^(final concentration of 1/100 HPS). Samples (50 μL) of each dilution were tested for haemolytic activity, using chicken erythrocytes (50 μL of a mixture of MBL-deficient serum and 10^9 ^chicken erythrocytes in VSB^2+^). The microtiter plates were placed in a water bath at 37°C for 1 h and then centrifuged for 10 min, 2500 rpm. Supernatant of each sample was transferred to a flat-bottom plate containing 200 μL Super Q per well. Haemoglobin release was measured in an ELISA reader at 405 nm. Percentages of haemolysis were calculated using controls for 100% (water lysed) and 0% (buffer incubated) haemolysis. After incubation, the degree of bystander erythrocyte lysis was translated into the number of active sites per erythrocyte (Z-value) using the equation of Borsos and Rapp [29]. Titters were read at Z = 0.300. To measure the alternative pathway activation by the different microorganisms, the experiment s described above was performed with HPS in EGTA-VB (8 mM ethylene glycol bis-(β-aminoethyl ether)-*N,N,N',N'*-tetraacetic acit with 2.5 mM Mg ^2+^) preventing classical and lectin pathway activation by removing Ca^2+^. Direct haemolysis of the erythrocytes by microbial products was excluded by incubating microorganisms and erythrocytes together with MBL-deficient serum only. All experiments were repeated at least three times

### Statistical analysis

Statistical significance was determined by unpaired Student's *t *test, using GraphPad Software program (Prism 5; GraphPad Software, Inc., San Diego, Calif.). *P *values of < 0.05 were considered to be statistically significant.

## Results

### 3.1. Binding of MBL to different pathogens

**Figure 1:**
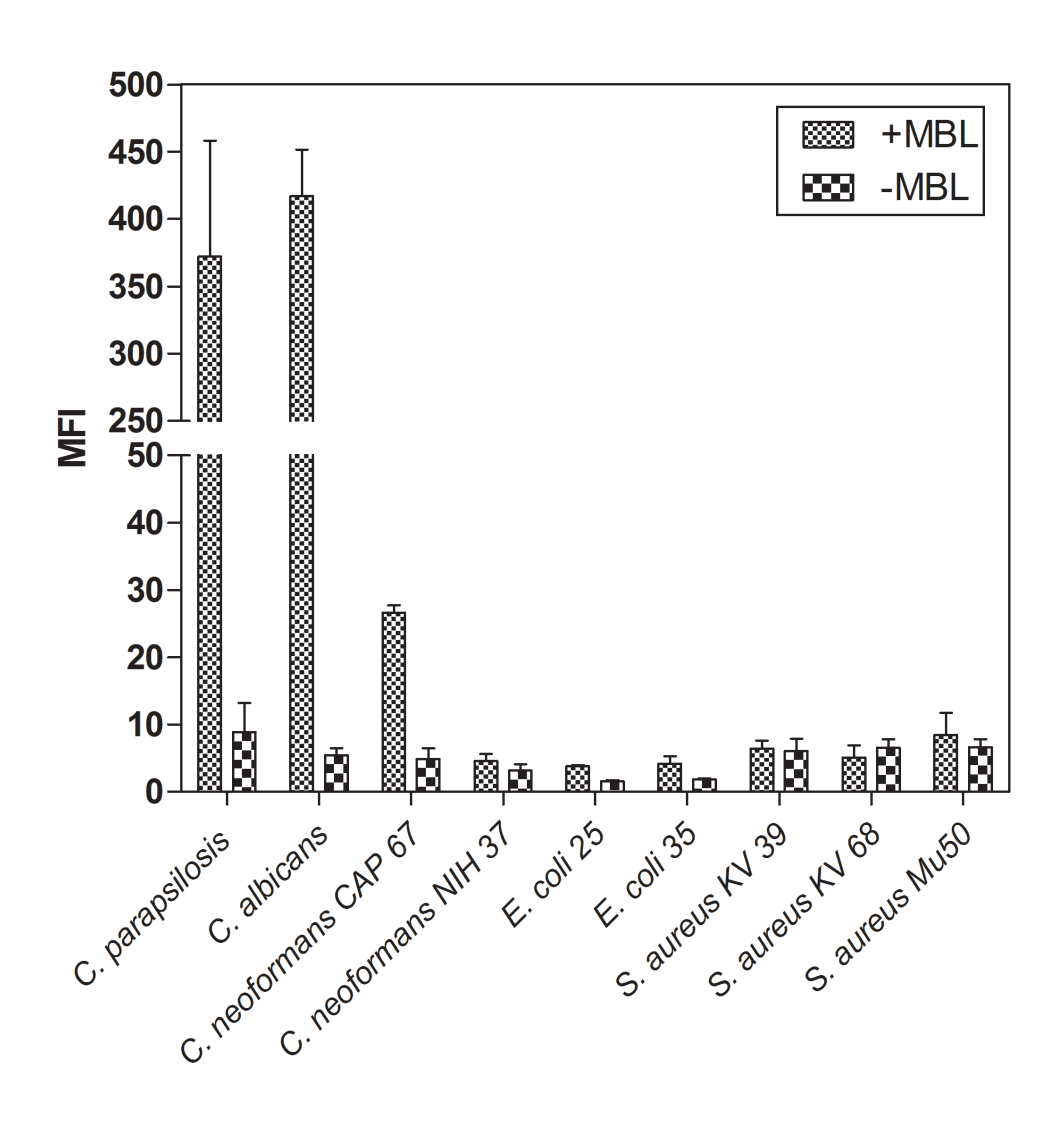
**Ca2+-dependent interaction between mannose-binding lectin (MBL) and *C. parapsilosis *and reference strains *C. albicans*, *C. neoformans*, *S. aureus *and *E. coli***. Microorganisms were incubated in the presence of 5 μg/mL purified human MBL in VSB^2+^. After incubation with mouse anti-MBL monoclonal antibody and FITC-labeled goat anti mouse IgG, binding of MBL to these microorganisms was analyzed by flow cytometry. Results are expressed as median fluorescence intensity (MFI). Data are the mean ± SEM of 3 separate experiments. Solid bars, addition of human purified MBL; open bars, no addition of human purified MBL.

A striking difference in binding patterns of MBL to the different microorganisms was found (Figure 1). Binding of MBL to *C. albicans*, *C. parapsilosis *and in a lesser extend to acapsular *C. neoformans *was found. Almost no binding of MBL was observed to *S. aureus*, *E. coli *and encapsulated *C. neoformans*. No binding was observed in the absence of Ca^2+ ^(data not shown) and when purified MBL was not added.

### C4 and C3b deposition on the various pathogens

**Figure 2:**
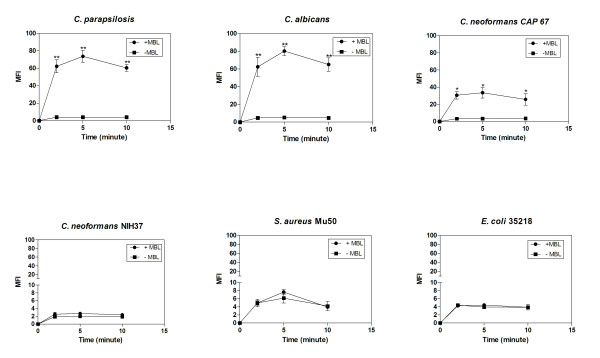
**Time-dependent C4 deposition on *C. parapsilosis *and reference strains *C. albicans*, *C. neoformans*, *S. aureus *and *E. coli *in MBL-deficient serum supplemented with exogenous purified human MBL**. Microorganisms were incubated with 10% MBL-deficient serum with or without 2.5 μg/mL purified human MBL, at 37°C over a time course of 10 min. Results are expressed as median fluorescence (MFI). Data are the mean ± SEM of 3 separate experiments. *P < 0.05 and ** *P*< 0.005 unpaired Student's *t *test of MFI.

With *C. albicans*, *C. parapsilosis *and of acapsular *C. neoformans *deposition of the cleavages product C4 was detected after 2 min of incubation in MBL-deficient serum and was significantly enhanced by the addition of MBL at 2 min up to 10 min (*P *< 0.005 or *P *< 0.05, unpaired Student' t test of MFI) (Figure 2). Deposition of C4 was maximal at 5 min, whereas in the absence of MBL C4 deposition attained its maximal amount slowly over the time course measured. In contrast, addition of purified MBL to MBL-deficient serum did not increase C4 deposition significantly to the capsulated strains of *C. neoformans*, *S. aureus *and *E. coli*.

**Figure 3:**
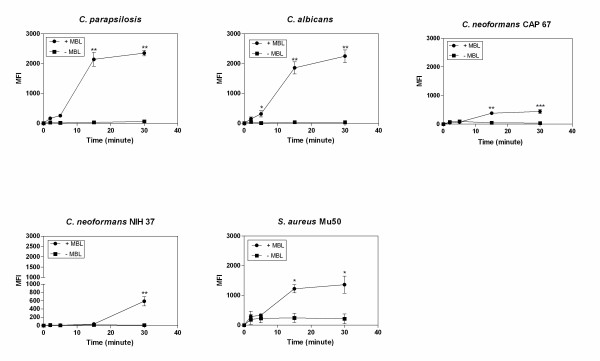
**Time-dependent C3b deposition on *C. parapsilosis *and reference strains *C. albicans*, *C. neoformans*, *S. aureus *and *E. coli *in MBL-deficient serum supplemented with exogenous purified human MBL**. Microorganisms were incubated with 10% MBL-deficient serum with or without 2.5 μg/mL purified human MBL, at 37°C for 30 min. Results are expressed as median fluorescence (MFI). Data are the mean ± SEM of 3 separate experiments. *P < 0.05 and ** *P*< 0.005 unpaired Student's *t *test of MFI.

Experiments with antibody to C3b (anti-C3b) showed that the addition of purified human MBL enhanced the deposition of C3b on *Candida *species and, to a lesser degree, C3b deposition on acapsular, encapsulated *C. neoformans *and *S. aureus *(Figure 3). With *Candida *species, acapsular *C. neoformans *and *S. aureus*, C3b deposition was detected after 2 min of incubation in serum, with enhancement of deposition in the presence of MBL, reaching significance at 5 up to 30 min for *C. albicans *(*P *< 0.05 unpaired Student' *t *test) and at 15 and 30 min for *C. parapsilosis*, acapsular *C. neoformans *and *S. aureus *(*P *< 0.005 or *P *< 0.05 unpaired Student' *t *test). An increase in C3b deposition was observed for capsulated *C. neoformans*, reaching significance only at 30 min (*P *< 0.05 unpaired Student' *t *test).

### Opsonophagocytosis of different pathogens

**Figure 4:**
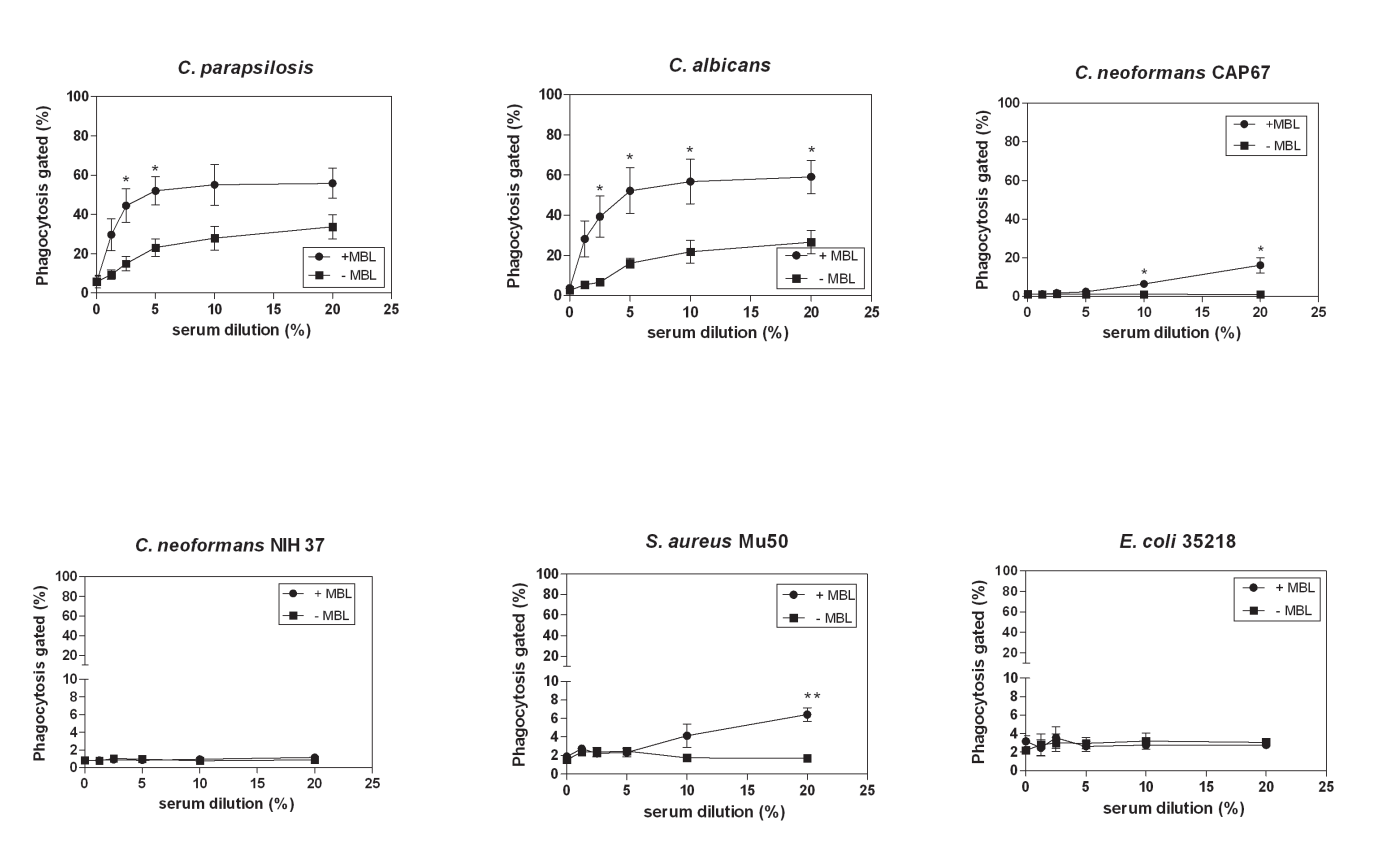
**Opsonophagocytosis of *C. parapsilosis *and reference strains *C. albicans*, *C. neoformans*, *S. aureus *and *E. coli *by human polymorphonuclear (PMN) cells**. Microorganisms were labelled with fluorescein isothiocyanate (FITC) and preincubated in the absence (control) or presence of 20% mannose-binding lectin (MBL)-deficient serum supplemented with 5 μg/mL human purified MBL, for 20 min. The yeast:phagocyte ratio was 2:1. Phagocytosis was analyzed by the use of flow cytometry and expressed as percentage of microorganisms-ingested PMN. Data are the mean ± SEM of 3 separate experiments. *P < 0.05 and ** *P*< 0.005 unpaired Student's *t *test of phagocytosis % gated.

Opsonophagocytosis of *C. albicans *and *C. parapsilosis *was enhanced by preincubation in the presence with purified MBL compared to preincubation with MBL-deficient serum alone, reaching significance at 2.5% up to 20% serum for *C. albicans *(*P *< 0.05, unpaired Student' *t *test of % gated) and at 2.5% and 5% serum for *C. parapsilosis *(*P *< 0.05, unpaired Student' *t *test of % gated) (Figure 4).

We noted less opsonophagocytosis in the presence with purified human MBL and the acapsular strain of *C. neoformans *compared to *Candida spp*., although significance was reached at 10% and 20% serum (*P *< 0.05, unpaired Student' *t *test of % gated). The capsulated strain of *C. neoformans *showed no opsonophagocytosis with or without adding purified human MBL. *S. aureus *and *E. coli *showed no opsonophagocytosis in the lowest concentration with or without adding purified human MBL. But MBL appears to play a role in the higher serum concentrations, reaching a significance at 20% serum for *S. aureus *(*P *< 0.005, unpaired Student' *t *test of % gated). When downstream complement components were inactivated by heating at 56°C, opsonophagocytosis was eliminated under all conditions tested (data not shown).

### Haemolytic assessment of complement activity via the lectin pathway

The haemolytic assay was used to characterize the different microorganisms as a weak or potent activator of the MBL arm of the complement system at a 1/32 dilution of HPS, since no activation of the alternative pathway was observed with this dilution (data not shown). In this assay, the number of microbes and the MBL concentration were varied. Addition of the inhibitory antibody directed against C1q eliminated the contribution of the classical pathway. The following organisms were ranked, in order of decreasing MBL-activating ability, *S. cerevisiae*, *C. albicans*, *C. parapsilosis*, encapsulated, and acapsular strain of *C. neoformans *and *S. aureus *and *E. coli *(Table 1). The *C. neoformans *strains, *S. aureus *strains, *E. coli *strains did not activate MBL at this serum concentration.

**Table 1:** Differential lectin pathway activating properties of *S. cerevisiae*, *C. albicans*, *C. parapsilosis*, *C. neoformans*, *E. coli *and *S. aureus *via MBL

**Microbial strain**	**Concentration at Z = 0.3 (CFU/well)**
*S. cerevisiae*	3.39 × 10^5^

*C. albicans*	4.0 × 10^5^

*C. parapsilosis*	4.9 × 10^6^

*C. neoformans encapsulated*	> 1 × 10^7^

*C. neoformans acapsular*	> 1 × 10^7^

*E. coli*	> 1 × 10^8^

*S. aureus*	> 1 × 10^8^

Z value stands for the amount of haemolysis in the haemolytic assay, a measure of the mean number of MBL activating sites per chicken erythrocyte. CFU: Colony-forming units

## Discussion

Mannose binding lectin (MBL), is a calcium-dependent plasma lectin that binds a wide range of microorganisms [5]. In the present study, we evaluated the role of binding of MBL at the subsequent deposition of C4 and C3b on the microbial cell wall. Also the role of MBL in opsonophagocytosis by PMN was studied. Well-characterized *C. parapsilosis *strain, *C. albicans*, *E. coli*, *S. aureus *and *C. neoformans *were used. These data demonstrate that MBL binds to *C. parapsilosis*, *C. albicans *and acapsular *C. neoformans*. MBL binding leads to activation of the lectin pathway of complement, demonstrated by deposition of C4 and C3 fragments and to enhanced opsonophagocytosis by PMNs. MBL is an important opsonin for phagocytosis of *Candida *species and acapsular *C. neoformans*. It lacks the function as an opsonin for phagocytosis of encapsulated *C. neoformans*. Indeed, MBL plays a much less important role in the process of opsonisation of the *S. aureus *and *E coli *strains used. The lack of MBL activation of *S. aureus*, *E. coli *and *C. neoformans *could be advantageous to the organism in allowing it to remain hidden from the MBL arm of the complement system (i.e., protected from lectin pathway-induced complement activation). Serum MBL levels would be unlikely to influence these organisms colonization/infection, compared to the yeast, which are strong activators of the MBL arm of the complement system. These data suggest the importance of MBL in the first-line defence against *Candida *species and acapsular *C. neoformans*.

Previously, studies have shown that MBL binds with high avidity to *C. albicans *and *C. parapsilosis *as well as to encapsulated *C. neoformans*, through mannan, a major component of fungal cell walls [4,30,31]. Microorganisms, as observed in the present study, fall into three groups. *S. aureus*, *E. coli *and encapsulated *C. neoformans *did not bind to MBL, acapsular *C. neoformans *bound MBL only weakly and both *Candida *species showed strong MBL binding. MBL has recently been shown to bind to *C. albicans *via its lectin domain, resulting in fungi agglutination on their hyphea outgrowths [9].

Capsules have an important role in protecting organisms *in vivo *against complement attack, by making them resistant to phagocytosis [13,32]. The acapsular form of *C. neoformans *has exposed carbohydrate residues on its surface that are suitable for interaction with lectin-like receptors [30]. Thus, the levels of MBL required for opsonophagocytosis may depend on the availability of binding epitopes on the infectious agents.

The opsonophagocytosis assay and the complement-deficient sera used in these experiments allowed us to measure MBL dependent opsonization, because the classical pathway was blocked with anti-C1q [7]. MBL increased the uptake of *C. albicans, C. parapsilosis *andacapsular *C. neoformans *by PMNs in serum. Since phagocytosis was not observed by binding of MBL in the absence of down-stream complement factors (data not shown), phagocytosis was enhanced via C3b-dependent opsonization recognized by complement receptors on PMNs. Thus, MBL is an opsonin only in the presence of complement.

In contrast to our results, Ip and Lau [9], using dendritic cells, reported that MBL binding does not lead to opsonophagocytosis, possibly due to the interference of MBL with the recognition of *C. albicans *by C-type receptors on dendritic cells, which mediate phagocytosis. Neth et al. [33] demonstrated that an MBL-mediated increases in opsonic C3 fragments enhanced opsonophagocytosis of *S. aureus *by neutrophils. However, Cunnion et al. [34] showed similar to us, that MBL-mediated complement activation, did not enhance *S. aureus *phagocytosis. They used hypo-γ-globulin serum, which had been affinity-depleted of MBL, whereas Neth et al. [33] used serum from adult individuals who were genetically deficient in MBL, but which could contain immunoglobulins. Bacterial strain differences could account for the differences find in these reports [4]. Comparable to our results with *E. coli*, previous experiments also have shown that MBL contributed little to opsonophagocytosis of gram-negative microorganisms [35-37]. Recently, Brouwer et al. [38] reported MBL binding on the surface of *S. pneumoniae*, *S. aureus *and *E. coli*, but did not observe any significant contribution of MBL to opsonophagocytosis of these organisms. Comparable to our results, Brouwer et al. [38] indicates that the lectin pathway of complement activation did not contribute to a large extent of the opsonophagocytosis of these bacteria.

Also it was shown that in MBL transgenic mice MBL plays an important role in the innate immunity [39]. In contrast to our observations, Shi et al. [40] reported that MBL-initiated opsonophagocytosis by both neutrophils and macrophages is an important first-line host defence against *S. aureus *in mice. Mice that do not have a functional MBL complement pathway are highly susceptible to infection with *S. aureus*. In that study, decreased phagocytosis of *S. aureus *by peritoneal macrophages in MBL-null mice was reported [40]. However, we only did *in vitro *studies, which may or may not explain the susceptibilities of MBL deficient individuals to these organisms. Further studies are needed to define the role of MBL in the defence against these bacteria.

Previously, it has shown, that *Neisseria meningitidis*, the causative agent of meningococcal disease, is a strong activator of MBL [3]. This is in line with clinical studies, which showed that MBL is associated with an increased risk of mucosal acquired infections including meningococcal disease [41]. However, the role of MBL as an opsonin may thus critically depend on the microbial species involved, interspecies variation and the type of phagocytes present.

A number of clinical studies have reported that MBL deficiency predisposes to *Candida *infections. Recently, Till et al. [42] described that patients with peritonitis with an early abdominal yeast infection, most commonly caused by *C. albicans *and *C. parapsilosis *[43], had lower MBL plasma levels than patients without such abdominal yeast infections. The incidence of abdominal yeast infections in patient with MBL variant genotype was significantly higher to those with no MBL variant genotype [42].

MBL present in the vaginal cavity has been found to act as recognition molecules for *C. albicans *that colonize the cervicovaginal mucosa, which suggest that the lectin pathway plays an important role against *Candida *infection [44]. Low levels of vaginal MBL in patients with recurrent vulvovaginal candidiasis (VVC) might predispose to *Candida *infections [45]. It has been proposed that MBL activity is critical in early life, when maternally acquired protection is decreasing and actively acquired immunity is still low [5,39,46]. MBL plasma concentrations at birth may be low due to both gene-polymorphisms and younger gestational age [47-49]. Thus MBL activity may play an important role in innate defence of *C. parapsilosis *in premature babies.

## Conclusion

In conclusion, the present study demonstrated the important role of MBL-mediated complement activation in opsonophagocytosis of *C. parapsilosis, C. albicans *and acapsular *C. neoformans*. MBL enhances opsonization of *C. parapsilosis*, *C. albicans *and acapsular *C. neoformans*, via the lectin pathway, which depends on the presence and availability of MBL binding epitopes. The binding of MBL by these yeasts and subsequent complement activation and opsonophagocytosis observed in our study may explain the observed increased risk of infections caused by these microorganisms in MBL-deficient individuals.

## Authors' contributions

AE participated in the design of the study, carried out the experiments and drafted the manuscript; HA participated in the design of the study and coordination; SJ carried out the experiment; HB participated in the design of the haemolytic assay; VJ conceived of the study, participated in its design and coordination. All authors read and approved the final manuscript.
